# Impact of water quality on Chronic Kidney Disease of unknown etiology (CKDu) in Thunukkai Division in Mullaitivu District, Sri Lanka

**DOI:** 10.1186/s12882-020-02157-1

**Published:** 2020-11-25

**Authors:** Kalaivani Gobalarajah, Prabagar Subramaniam, Uthpala Apekshani Jayawardena, Gobalarajah Rasiah, Sittampalam Rajendra, Jasotha Prabagar

**Affiliations:** 1 Centre for Environmental Studies and Sustainable Development, The Open University of Sri Lanka, Colombo, Sri Lanka; 2 Industrial Technology Institute, 363, Bauddhaloka Mawatha, Colombo-7, Sri Lanka; 3 Department of Zoology, Faculty of Natural Sciences, The Open University of Sri Lanka, Colombo, Sri Lanka; 4 Department of Construction Technology, University College, Jaffna, Sri Lanka; 5 Department of Surgery, Faculty of Medicine, University of Jaffna, Jaffna, Sri Lanka; 6 Department of Chemistry, Faculty of Science, University of Jaffna, Jaffna, Sri Lanka

**Keywords:** Chronic kidney disease of unknown etiology, Water quality parameters, Serum creatinine

## Abstract

**Background:**

Increase in the number of cases in Chronic Kidney Disease of Unknown etiology (CKDu) in Sri Lanka has become a health issue of national concern. Even though, Northern Province is not identified as a high-risk province, there is an increasing trend of CKDu after the end of civil war in the Northern Province.

**Methods:**

The present study was conducted in Thunukkai Division in Mullaitivu District to investigate the socio demographic and clinical pattern of CKDu patients and to evaluate the quality of their water sources. The samples were selected by using stratified purposive random sampling method which represented 29% of total CKDu patients in Thunukkai Division. Pretested structured questionnaire was administered to collect the data from the CKDu patients. The association between serum creatinine excreted by CKDu patients and the water quality parameters were determined by using linear regression model.

**Results:**

Among the patients, 80% were male with over 68% falling in the age range of 50–70. Majority (90%) were involved in agriculture related occupation. Smoking and alcohol consumption were detected as common habits among 40% of the patients. Secondarily developed, hypertension (60%) and diabetes (34%) were reported as common diseases in the area. Dug wells served as the commonest source of drinking water in the area (90% households) together with few tube wells. Physicochemistry of more than 50% of the water samples revealed higher electric conductivity, salinity, total dissolved solids, total hardness and Na levels compared to drinking water standards in Sri Lanka.

**Conclusions:**

Serum creatinine levels of the CKDu patients were significantly and negatively correlated with phosphate while positively correlated with total dissolved solids (TDS) and arsenic content of the drinking water. Geospatial mapping of TDS and arsenic in drinking water with the occurrence of higher serum creatinine levels confirmed the same trend. Thus, the total dissolved solids and arsenic in drinking water may have positive correlation with the occurrence of CKDu in Thunukkai region in the Mullaitivu District of Sri Lanka.

## Background

Chronic Kidney Disease of unknown etiology (CKDu) is the occurrence of Chronic Kidney Disease (CKD) without a known underlying cause [1]. Since its first report in mid-90’s cases of CKDu have increased tremendously in North Central Province of Sri Lanka [2]. It is estimated that thousands of Sri Lankan people are affected by CKDu, mostly poor families living in remote areas. However, the number of CKDu patients and causes of the disease are unknown. Unfortunately, the research studies conducted to date were unable to provide exact cause/s of CKDu. A common conclusion is that the CKDu is caused by multiple factors involving environmental and social impacts [3].

There are several etiologies, proposed by the researchers of CKDu, including demographic factors of the affected community [4], quality of their drinking water including hardness [5], agrochemical and heavy metal contaminations [5, 6] fluoride level [7], the genetic makeup of vulnerable populations [8], etc. Demographic factors include the socioeconomic characters of a population such as age, sex, occupation etc. Several studies conducted in the North Central Province revealed that the main livelihood of CKDu affected population is farming and the age of the patients ranged between 30 and 60 with higher prevalence among elderly males over 50 years of age [2, 5, 9]. Low water consumption during farming activities and dehydration due to the exposure to direct sunlight may have led to renal failure [5]. Disease aggravating habits such as alcohol consumption, betel chewing and smoking have also been investigated in relation to patient demography [5]. A common genetic variant close to SLC13A3 is reported to be related to CKDu [10]. This has been identified as the most sensitive gene marker to predict the renal disease of type 2 diabetes mellitus. Furthermore, genes such as IGFBP1, KIM1, GCLC and GSTM1 are proposed to be used in combination for early determination of CKDu [11].

Correlation between high groundwater hardness and the occurrence of CKDu have been frequently reported [8, 9, 12, 13]. According to World Health Organization (WHO) hard water is mainly caused by the presence of calcium, magnesium, strontium, and iron together with carbonate, bicarbonate, sulphate and chloride anions. Furthermore, possible correlation between fluoride (F) in drinking water and the prevalence of CKDu was suggested in various instances [12–14]. According to WHO (2011), Sri Lanka is one of the tropical countries in the world with higher fluoride content in water resources, reaching the upper limit value of 0.6 mg/L [15]. In most of the CKDu endemic areas F content exceeds the upper limit value [16]. Maximum permissible contaminant level of Arsenic (As) is 10 μg/L though As contamination in the disease endemic regions exceeded the upper limit [17]. However, Balasooriya et al., [9] and Nanayakkara et al., [8] found insignificant levels of As and other trace elements in drinking water of CKDu endemic areas of Sri Lanka.

Algal toxins have also been considered as a suspect of the CKDu [18]. According to WHO, eighteen different types of cyanobacteria are capable of producing toxins under favourable conditions. Among them, fifteen toxic producing cyanobactria have been identified in Sri Lankan reservoirs and canals. These toxins are identified as hepatotoxic, dermatotoxic, neurotoxic and nephrotoxic compounds [18].

CKDu has direct impact on patients’ lives including their livelihood activities. As the disease advances, patients become too ill to continue their employment, affecting the economic conditions and wellbeing of the entire family.

**Fig. 1:**
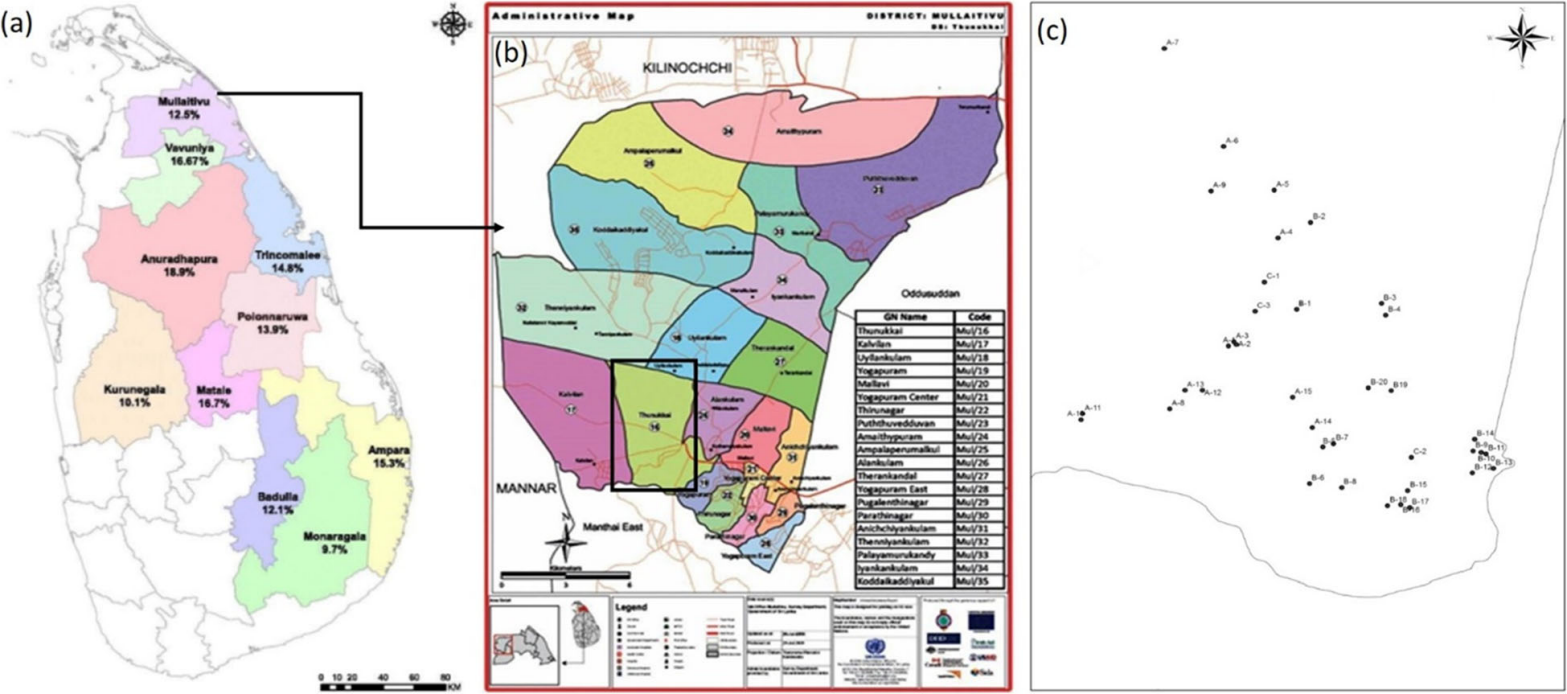
Thunukkai Divisional secretariat of the Mullaitivu District showing the distribution of drinking water sources (sampling points, A1-A15, B1-B20) of the patient households and control samples from C1-C3. {**a** CKD prevalence rates across the most affected districts in Sri Lanka [19], **b** Administrative map of the Mullaitivu District (Sri Lanka Surveys Department, Maps and Geoinformations, https://www.survey.gov.lk) **c** Sampling points in Thunukkai DS (Drawn with ArcGIS 10.1, https://www.arcgis.com/index.html)}

Current data on CKDu distribution show the occurrence of the disease in North Central, North Western, Southern, Eastern and Uva Provinces. Even though, Northern Province is not identified as a high-risk province, CKDu is developing at an alarming rate after the end of civil war in Northern part of this country. This may be the result of increased use of agrochemicals, residuals of explosives and newly emerging industries with unplanned effluent disposal leading to aquatic pollution in natural reservoirs. Northern Province comprises of five districts; Jaffna, Killinochchi, Mullaitivu, Vavuniya and Mannar. Among these Mullaitivu and Vavuniya have been considered “at risk” for the occurrence of CKDu with 09 other districts from North Central, Central and Uva provinces [19, 20] (Fig. 1). And Northern Province appear to have higher CKD prevalence than Central or Southern Provinces [20]. In the present study, Thunukkai of Mullaitivu District was selected as its prevalence of CKDu has not been studied much. Most of the people in Thunukkai are farmers, who carried out paddy farming throughout the year. Majority in Thunukkai Divisional secretariat use shallow dug wells and reservoirs for their daily consumption without any treatment of water. Thunukkai Divisional Secretariat has many reservoirs. Some GN (“Grama Niladhari”-Public Service Officer) divisions are named according to the name of reservoirs such as Anichakulam, Thenniyankulam, Kodaikadiyakulam etc.

Objectives of this study were to analyze the socio demographic and clinical pattern of CKDu patients in Thunukkai Division, and to determine the water quality parameters, such as cadmium (Cd), arsenic (As), nitrate, phosphate, fluoride, hardness, total dissolved solids (TDS), pH, sodium (Na), potassium (K) and electric conductivity of the drinking water in the wells and to assess correlations between water quality parameters and the serum creatinine levels of the CKDu patients.

## Methods

Ethical clearance was obtained from the Ethics Review Committee, Faculty of Medicine, University of Jaffna. Data regarding CKDu patients were gathered during the period of January 2018 to July 2018 from respective Regional Director of Health Service in Northern Province. In Mullaitivu district, 631 cases of both CKD, including CKDu were identified in 3 Medical Office of Health (MOH) which are Thunukkai (120 cases), Manthai East (86 cases) and Sampathnuwara (425 cases). Thunukkai division was selected for the present study as it is a scarcely studied area, affected by the civil war.

In Thunukkai Division 120 patients, out of the total population of 10,172 were identified as CKD/CKDu patients by MOH office Mallavi. The water samples were collected by using stratified purposive random sampling method which represented 29% of total CKDu patients in Thunukkai Division. Sample size (n) was estimated using the equation, n = Z^2^ p(1-p)/ e^2^, where Z = confidence level at 95% (i.e. 1.96), *p* = estimated prevalence of the area (120/10,172 = 0.0118) and e^2^ = margin of error. Even though, the sample size *n* = 18 was statistically sufficient 35 patients were randomly sampled to cover all the CKDu positive villages of the area.

Sociodemographic data were collected through an interviewer administered questionnaire, developed for the study and filled during the visit to patient’s houses (Supplementary file). Data include age, gender, occupation of the patient, clinical signs and symptoms of CKDu (serum creatinine level) and potential habits for the disease progression (smoking and alcohol consumption).

As CKDu patients were distributed in 65% of total villages in Thunukkai Division water samples were collected in these villages. A total of 38 water samples; 35 samples from CKDu affected areas (Fig. 1, A1-A15, B1-B20) and 3 control samples (Fig. 1, C1-C3) were collected in August 2018. The three control samples were collected from places in Thunukkai central, Yokapuram west and Ugilankulam, where there were no records of CKDu patients.

Water samples were collected during the dry season. Before collecting water, the water column was thoroughly stirred with the collecting bucket. If the water surface has floating scum or algae, those were skimmed before collecting the samples. Water was collected over the depth of 10 cm below the water surface. In the case of tube wells, pumps were used to take samples of water. Water samples were collected in cleaned plastic bottles which were then refrigerated at 4 °C until assaying. Physicochemical parameters such as, turbidity, colour, odour, total dissolved solids, alkalinity, salinity, electrical conductivity, NO_3_^−^, PO_4_^3−^, SO_4_^2−^, F^−^, total hardness, Ca^2+^, Mg^2+^ and Cl^−^ of the water samples were measured within a week.

Physicochemical properties of the water samples were determined with standard instruments, following the standard procedures. Onsite measurements were obtained with portable meters for pH (Jenway Phm6, UK), turbidity, electrical conductivity and salinity (Senso direct, con110, USA). TDS was measured gravimetrically with HCl acid. Alkalinity and total hardness were measured using sulfuric acid based and EDTA based complexometric titration methods, respectively. Fluoride (F^−^) was measured with SPANDS Spectrophotometric method. NO_3_^−^, PO_4_^3−^, SO_4_^2−^ ions were determined by COD multiparameter photometer (HI83399, UK). These analyses were carried out in the Department of Chemistry, University of Jaffna and As and Cd concentrations were measured in Industrial Technological Institute in Colombo using graphite furnace atomic absorption spectrophotometry (GFAAS) with a precision of 0.001.

By using Geographic information system (GIS) software (ArcGIS 10.1) mapping was conducted for nitrate, phosphate, total hardness, total dissolved solid, fluoride and Arsenic content in water.

Physicochemical parameters of the test samples were compared with those of the control samples and the Sri Lankan standards for potable water. To assess the correlation between water quality parameters and serum creatinine levels of the CKDu patients, linear regression, model was used. Through this analysis, relationships between the target variable (dependent variable) and a set of independent variables (covariates) were quantified. The regression equation estimates a coefficient for each variable. The goal of regression analysis is to generate the line that best fits the observations. However, the best fitted line for the data leaves the least amount of unexplained variation, such as the dispersion of observed points around the line. The following formula describes the linear relationship between dependent and independent variables.

$$\mathrm{Y}=\upbeta 0+\upbeta 1\\mathrm{a}+\upbeta 2\mathrm{b}+\upbeta 3\\mathrm{c}+\upbeta 4\\mathrm{d}+\upbeta 5\\mathrm{e}+\upbeta 6\\mathrm{f}+\mathrm{error}$$

Where, dependent variable (Y) is the serum creatinine levels of the CKDu patient. Independent variables; a-nitrate, b-fluoride, c- phosphate, d- Total Dissolved Solid, e- Total hardness, f- Arsenic content in water. Intercept β0- is a constant that defines where the linear trend line intercepts the Y-axis. Coefficient β1, β2, β3, β4, β5 and β6; constants that represent the rate of change in the dependent variable as a function of changes in the independent variable. It is the slope of the linear line. Error, represents the unexplained variation in the target variable. It is treated as a random variable that picks up all the variation in Y that is not explained by X.

## Results

### Demography of the CKDu patients

Ages of CKDu patients in Thunukkai Division ranged between 30 and 80 years. Among the studied patients 24 were in the 50–70 years range, comprising 63% of the total sample (Table 1). CKDu was more prevalent among males as the male to female ratio was 4:1. Among them 90% were engaged in agriculture related occupation while the rest were laborers, drivers, or unemployed people.

**Table 1:** Demographic data and baseline characteristics of the CKDu patients in Thunukkai, Mullaitivu District, Sri Lanka

Data	Number of patients (%)
**Age distribution**	
30–40	4 (11.42%)
41–50	5 (14.28%)
51–60	12 (34.28%)
61–70	12 (34.28%)
71–80	2 (5.71%)
**Sex**	
Male	28 (80%)
Female	07 (20%)
**Occupation**	
Agriculture related	31 (90%)
Other	02 (5%)
No occupation	02 (5%)
**Impact of other habits**	
Smoking	09 (25.71%)
Alcohol consumption	07 (20%)
Both- smoking & alcohol	07 (20%)
**Disease history**	
Hypertension	15 (42.82%)
Diabetes	06 (17.14%)
Both hypertension & diabetes	06 (17.14%)

According to the data obtained from the questionnaire, more than 20% of the patients either smoked or consumed alcohol while another 20% did both.

Clinical data of the selected patients showed existence of other non-communicable diseases such as hypertension (43%) and diabetes (17%) with 17% suffering from both the diseases. The disease history of the patients revealed that they were diagnosed with hypertension and diabetics secondarily, only after they developed CKDu. Furthermore, a spotty pigmentation which was similar to arsenic related keratosis was observed in the palms of three male patients of the study group.

### Physicochemical characters of the drinking water

Dug wells and tube wells serve as the major sources of drinking water in the study area. Thus, drinking water samples from 31 dug wells and 4 tube wells were collected and analysed for the physicochemical parameters. The results of the analyses are given in the Table 2. Among the parameters salinity, TDS and total hardness showed highest deviations with the corresponding values of more than 50% samples exceeding the relevant values of the Sri Lankan standards, SLS 614:2013 for drinking water (Salinity ranged between 0.13–3.66 g/L with an average of 0.69 g/L (standard error of mean, (SEM) = 0.12), with 50% of the samples exceeding the standard value of 0.5 g/L. Similarly, in 63% of the samples, TDS content exceeded the standard (400 mg/L) with an average value of 687 mg/L (SEM = 115) and reaching a maximum of 3570 mg/L. Total hardness of the samples ranged between 39.84–683.26 mg/L (SEM = 24.9) with 65% of the samples exceeding the standard (250 mg/L).

**Table 2:** Physicochemical properties of the water samples collected from Thunukkai, Mullaitivu District, Sri Lanka

Water quality parameter	Control (Mean ± SD^a^)	Samples			SLS^b^ 614 (2013)	% of water sample exceeding SLS value
		Max	Min	Mean (±SD)		
Turbidity (NTU^c^)	0.38 ± 0.09	26.5	0.3	2.3 ± 4.41	2	28.8
pH	8.0 ± 0.0	9.1	7.4	8.2 ± 0.4	6.5–8.5	17.1
Electric conductivity (μS/cm)	935.67 ± 238.7	6900	330	1413.31 ± 1275.4	750	17.1
Salinity (g/L)	0.416 ± 0.13	3.36	0.13	0.69 ± 0.70	0.5	50
TDS^d^ (mg/L)	418 ± 0	3750	136.3	687 ± 681	400	63.16
Nitrate (mg/L)	51.66 ± 31.50	295	0	28.13 ± 53.31	50	15.17
Fluoride (mg/L)	0.37 ± 0.24	22.3	0.1	1.73 ± 4.10	1	39.5
Phosphate(mg/L)	3.1 ± 2.12	4.84	0.06	0.84 ± 0.64	2	10.6
Total hardness(mg/L)	340.2 ± 76.1	683.2	39.84	295.76 ± 147.52	250	65.4
Magnesium(mg/L)	29.28 ± 5.89	68.13	0	30.19 ± 1.87	50	18.42
Calcium(mg/L)	86.36 ± 22.98	193.22	3.98	64.074 ± 43.20	100	21.05
Sodium(mg/L)	7.2 ± 5.43	41.6	0.1	13.52 ± 11.46	200	0
Potassium(mg/L)	4.8 ± 4.16	9.6	1.3	3.75 ± 2.00	10	0
Sulphate(mg/L)	13.3 ± 7.63	160	0	37.10 ± 31.35	250	0
Alkalinity(mg/L)	6.37 ± 1.26	31.87	2.46	7.2496 ± 5.41	200	0
Chloride(mg/L)	184.96 ± 139.9	2066.53	6.95	367.27 ± 514.75	250	31.42
Arsenic (lowest detected value) (mg/L)	Less than 0.0001 ± 0	0.03	Less than 0.0001	0.0054 ± 0.0051	0.01	23.7
Cadmium (μg/L)	Not detected	Not detected			0.003	0

^a^Standrad deviation, ^b^Sri Lanka Standards, ^c^NTU- Nephelometric Turbidity Units, ^d^TDS-Total dissolved solids

Among other parameters, turbidity, fluoride, chloride, calcium and arsenic contents showed higher deviations from the SLS 614:2013, with more than 20% samples exceeding the respective limits. Mean turbidity of the drinking water samples was 2.3 NTU (Nephelometric Turbidity Unit) (SEM = 0.74) with a range of 0.3–26.5 NTU, all exceeding the mean turbidity of control samples. Ten water samples (29%) exceeded the standard turbidity level (2NTU-SLS 614:2013).

Fluoride content averaged 1.73 mg/L (0.1–22.3 mg/L) (SEM = 0.69), with 39% samples exceeding the standard of 1 mg/L. Similarly, chloride content of 31% of the water samples exceeded the standard value of 250 mg/L with an average of 367.27 mg/L (6.95–2066.53 mg/L) (SEM = 87.0). Calcium averaged 64.07 mg/L (3.98–193.22 mg/L) (SEM = 7.3) with 21% of the samples having higher values than the standard value (100 mg/L) for calcium in drinking water. Even though, arsenic detected only in nine samples, the concentration exceeded the standard value (0.01 mg/L) reaching as high as 0.03 mg/L in some water samples.

Water quality parameters such as pH, electric conductivity, nitrate, phosphate and magnesium contents showed no substantial deviations, with only 10% of the samples exceeding the SLS 614:2013. pH of the water samples was 8.2 on the average (SEM = 0.1), ranging between 7.4–9.1. Only six samples had a pH of more than 8.5 whereas no water sample had pH below 6.5. The mean value of electrical conductivity was 1416.31 μS/cm (SEM = 216), with the values ranging from 330 to 6690 μS/cm. 17% of the samples exceeded the standard (750 μS/cm). Nitrate content of the samples averaged 28.13 mg/L, (SEM = 9.01), with values ranging from 0 to 295 mg/L. Only 15% of the water samples contained higher nitrate levels than the standard value, 50 mg/L. The mean phosphate content was 0.84 mg/L (0.06–4.8 mg/L) (SEM = 0.11) with 10% of the samples exceeding the desirable level. Magnesium content had a mean value of 30.19 mg/L with 18% of samples exceeding the standard value.

All the other physicochemical parameters including, Na, K, sulphate contents and had lower levels compared to their respective Sri Lankan standards. Cadmium was not detected in any of the water samples collected from the sampling area.

**Fig. 2:**
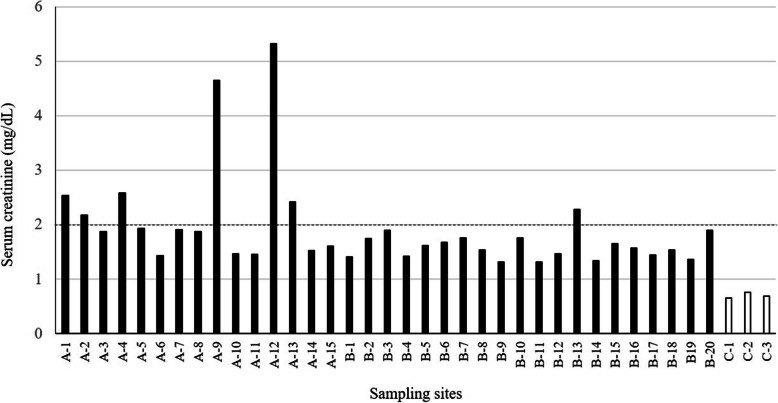
Serum creatinine data of CKDu patients, collected from different locations, A1-C2 in Thunukkai. A1-B20 are CKDu endemic areas, C1-C3 are none-CKDu areas. Reference line represents 2 mg/dL creatinine level

According to the data gathered through the questionnaire, the serum creatinine levels of CKDu patients of Thunukkai ranged between 1.31–5.32 mg/dL, with an average of 1.91 ± 0.84 (SEM = 0.14) mg/dL. Serum creatinine of the control samples had an average, of 0.7 ± 0.05 mg/dL (SEM = 0.03), as illustrated in Fig. 2, showing a significantly low value (t = 8.24, *p* = 0.0001).

### Correlation between water quality parameters and CKDu patients

The effect of physicochemical characters of water on the occurrence of CKDu was evaluated by applying the linear regression model, where serum creatinine concentration was treated as the response variable while water quality parameters (nitrate, fluoride, phosphate, etc.) were treated as the explanatory variables. Results are given in the Table 3 below.

**Table 3:** Regression analysis between serum creatinine of CKDu patient and explanatory variables, phosphate, TDS and Arsenic contents in water from Thunukkai, Mullaitivu District, Sri Lanka

Explanatory variables	Coefficient	St. Error	T value	***P*** value
(Intercept)	1.660e^+ 00^	1.767e^−01^	9.394	5.63e-11 ***
PO4^3−^	2.113e^−01^	1.009e^−01^	−2.095	0.0437 *
TDS	2.829e^−04^	1.592e^−04^	1.777	0.0845
As	1.065e^+ 02^	2.135e^+ 01^	4.986	1.79e-05 ***

*R*^2^ = 0.5109, *P<0.05 and ***P<0.01

**Fig. 3:**
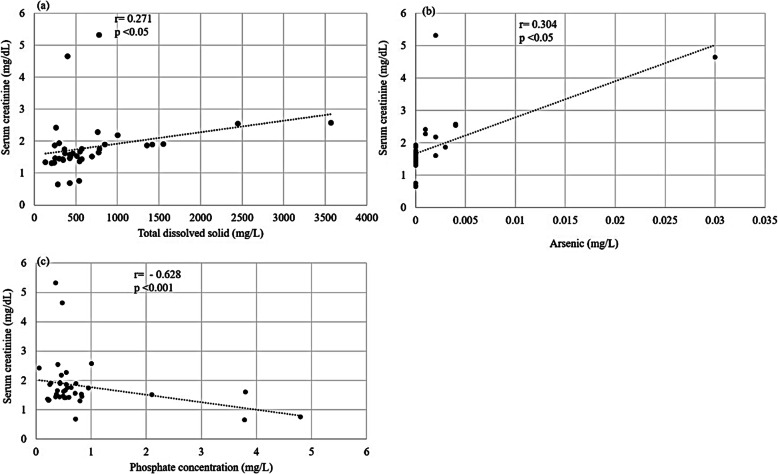
Scatter plots of total dissolved solids (TDS), arsenic and phosphate contents in drinking water showing significant correlations with the serum creatinine levels of the CKDu patients in Thunukkai

*R*^2^ value of 0.5109 suggests, that six explanatory variables; fluoride, phosphate, TDS, total hardness and arsenic, together account for about 51.09% of variation in the serum creatinine concentration of the CKDu patients. Among these physicochemical parameters, TDS and As contents showed significantly positive correlation (*p* < 0.05) with the creatinine levels while phosphate content showed significantly negative correlation (*p* < 0.001, Fig. 3). On the other hand, nitrate content in the drinking water showed no influence on serum creatinine of the CKDu patients in Thunukkai.

**Fig. 4:**
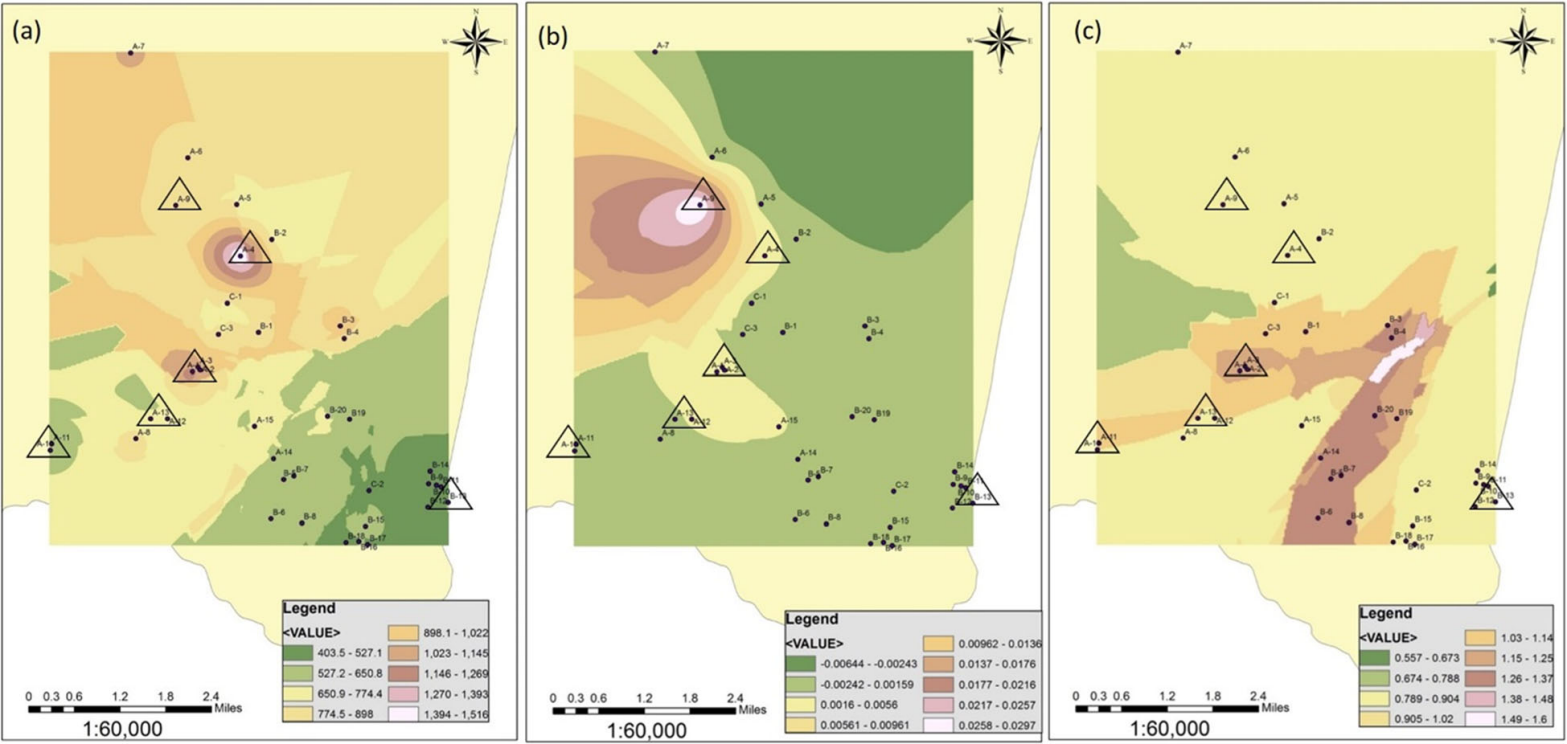
GIS mapping of **a** TDS-total dissolved solid, **b** arsenic and **c** phosphate distribution in the ground water in Thunukkai Division. Six triangles in each plot represent CKDu patients with higher serum creatinine content over 2 mg/dL. (Source: Created by the authors with ArcGIS 10.1, https://www.arcgis.com/index.html)

**Fig. 5:**
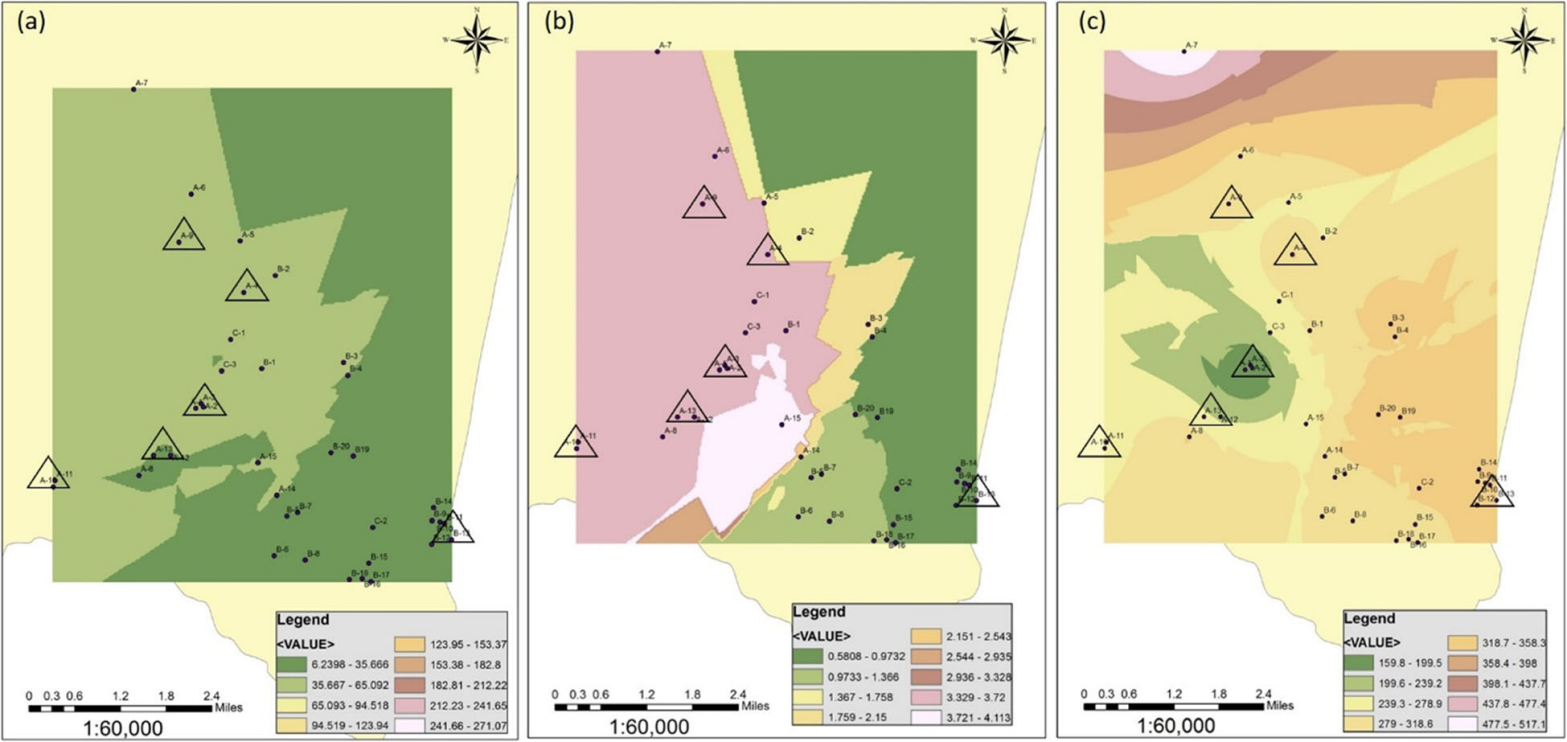
GIS mapping of **a** Nitrate, **b** fluoride and c total hardness content in the ground water in Thunukkai Division. Six triangles in each plot represent CKDu patients with higher serum creatinine content over 2 mg/dL. (Source: Created by the authors with ArcGIS 10.1, https://www.arcgis.com/index.html)

The measured total hardness, TDS, nitrate, fluoride, phosphate and arsenic contents in the drinking water of the study area are illustrated in Figs. 4 and 5. Analysis of the spatial distribution data of the patients indicated that seven sampling points have creatinine levels over 2 mg/dL in the order of, A12 > A9 > A4 > A1 > A13 > B13 > A2. When these locations were overlapped on the GIS mapping distinct interrelations were identified in TDS, As and phosphate with the creatinine levels (Fig. 4), agreeing with the trend observed in the regression analysis. Higher serum creatinine levels, over 2.5 mg/dl appeared to be linked with higher total dissolved solids content 650.9–1516 mg/L (Fig. 4a) and higher arsenic content 1.6–29.7 μg/L (WHO limit of As is 10 μg/L) (Fig. 4b) and low phosphate content, 0.789–0.904 mg/L (Fig. 4c). The spatial distribution of nitrate, fluoride and total hardness contents showed no direct influence on serum creatinine levels in the study samples (Fig. 5a, b & c).

## Discussion

The present study revealed that the male farmers, aged between 50 and 70 in Thunukkai Division of Mullaitivu developed higher risk of CKDu. Smoking and alcohol consumption may enhance the vulnerability towards the disease. The patients have developed hypertension and diabetes as secondary illnesses after developing the CKDu. Drinking water quality of the area was not at the desirable level, particularly with high ionic content leading to high salinity, dissolved solids and hardness levels. Secondarily, turbidity, fluoride, chloride, and magnesium levels reported disturbingly higher values compared to SL standards. As predicted by the regression model, fluoride, phosphate, TDS, total hardness and arsenic levels together accounted for considerable variation in the serum creatinine of the studied patients. This trend was further validated by the association of geospatial distribution of TDS, arsenic and phosphates with the occurrence of higher serum creatinine levels of the study samples.

Demographic data of the study comply with many other studies conducted in CKDu issue in Sri Lanka. Sex distribution of the presents study, i.e. 4:1, (Male: Female) was consistent with Wanigasuriya et.al [21]., Noble et.al [22]., Ranasinghe et al. [2], and Balasooriya et al. [9] which reported male preponderance. However, the age distribution was not compatible with Wanigasuriya et.al., [21]) and Noble et.al., [22] who reported as the majority of patients were in the 40–50 years range unlike the 50–70 range in the present study. On the other hand, many recent studies reported consistent findings where more than 60% of the CKDu patients were over 50 years of age [2, 12] Spotty pigmentations on palms, observed by Paranagama [23] was consistent with similar type of patches observed in the patients of Thunukkai in the current study. Dermatological data of these patients who used arsenic rich water suggested the development of early stage of arsenic related keratosis. However, a proper histopathological investigation should be conducted for the validation purposes.

Drinking water quality parameters were compatible with the groundwater quality data available for North Central province through various CKDu based research conducted to date. However, the total hardness, calcium and magnesium ion concentration, electric conductivity levels of the groundwater of Thunukkai were substantially high compared to those of the groundwater of North Central province [5, 17, 24, 25]. On the other hand, As and Cd in the groundwater of Thunukkai were low compared to the levels detected in the North Central province [5, 17]. Nevertheless, in agreement with the present study, Wickramarathna et al., [12] reported insignificant levels of Cd and As in groundwater of Girandurukotte, Wilgamuwa and Nikawewa areas. Chloride ion content in the groundwater of Thunukkai was high compared to the groundwater in CKDu high prevalent areas such as Padaviya, Kebithigollawa, Medawachchiya, & Kahatagasdigiliya, and moderate prevalence areas such as Mihintale, Talawa, and Nochchiyagama of the dry zone of Sri Lanka [26]. Similarly, fluoride levels were relatively high in Thunukkai compared to those of the dry zone of Sri Lanka as reported by Chandrajith et al., [27]. Water hardness and the conductivity levels in Thunukkai were compatible with those of the high and moderately prevalent areas [26].

Correlation data of the water quality parameters and serum creatinine levels revealed a significant negative relationship with phosphate and positive relationships with total dissolved solid (TDS) and arsenic content of the drinking water. This observation was further confirmed by geospatial mapping of the quality of the constituents and the occurrence of higher serum creatinine levels in study samples. Patients with extremely high levels of serum creatinine (over 4.5 mg/dL) appear to consume water from wells with higher TDS and arsenic contents. This higher availability of arsenic in ground water of Thenyiakulam (A9 site), Kalvilan (A12 site) Thunukkai (A4 site) may have related to the sediment characteristics of the area, where higher mobilization of arsenic ions occur through the availability of carbonate minerals of decaying organic matter which facilitate rapid release of arsenic ions from the As-adsorbed Fe-oxyanions in sediments [28, 29]. Thus, in future studies, analysis of sediment characteristics of the drinking water sources may be pivotal for understanding the overall process.

Similar to arsenic, several other constituents in drinking water and the diet were linked with the serum creatinine levels [30]. For example, complying with the present study nitrate in the diet was only slightly linked with serum creatinine of CKDu patients [31, 32]. In contrast to our results, significant association between the fluoride content and the occurrence of CKDu was revealed by several other researchers, including Illeperuma et.al., [7], Balasooriya et al., [9], Wickramarathna [12], Jayasinghe [33] and Wijeratne et.al., [34].

As given in the present study, phosphate ions in water may negatively influence the serum creatinine of CKDu patients. Thus, the negative sign of the coefficient implies that when phosphate content in the water increases, possibility of occurring CKDu decreases. The coefficient of phosphate shows that by holding nitrate, fluoride, total dissolved solid, total hardness and arsenic content, possibility to occur CKDu decreases by 0.2113 times for every unit increase in phosphate content. This result found to be consistent with Eddington et.al., [35]. Furthermore, total dissolved solids positively influence the serum creatinine levels of the study participants. The positive sign of the coefficient implies that when TDS content in the water increases, possibility to occur CKDu increases. The coefficient of TDS shows that by holding nitrate, fluoride, phosphate, total hardness and arsenic constant, possibility to occur CKDu increases by 0.2113 times for every unit increase in TDS content in water. On the other hand, total hardness showed no association with the serum creatinine levels, i.e. not compatible with Jayasumana et.al., [36] and Paranagama [37] who concluded positive and significant relationship between total hardness in water and CKDu. However, low *R*^2^ value obtained in the present study may affect the significance of the findings. Thus, further studies should be conducted with a higher sample number and a broader study area.

## Conclusion

The chronic kidney disease of unknown etiology in Thunukkai of the Mullaitivu District in the Northern Province of Sri Lanka showed male preponderance, M: F 4:1, at the age range of 50–70, revealing 80% occupational association with agricultural activities. Secondary development of hypertension and diabetes were observed in CKDu patients. Spotty pigmentation, similar to arsenic related keratosis was observed in the palms of the patients, lived in the areas where detectable arsenic levels in their drinking water.

Evaluation of drinking water revealed substantially high ionic content leading to higher electric conductivity, salinity, total dissolved solids and total hardness levels compared to those of the Sri Lankan standards. Serum creatinine levels of the CKDu patients were significantly and negatively correlated with phosphate and positive correlation with arsenic and TDS contents, contributing more than 50% variation in serum creatinine. The results may be concluded that water quality parameters such as phosphate, total dissolved solid and arsenic content are significantly correlated with CKDu in Thunukkai of Mullaitivu District in Sri Lanka.

## Supplementary Information

**Additional file 1.**

## Data Availability

The datasets used and/or analysed during the current study are available from the corresponding author on reasonable request. Additional data are available as a supplementary file.

## Abbreviations

COD: Chemical Oxygen Demand
CKDu: Chronic Kidney Disease of unknown etiology
EDTA: Ethylenediaminetetraacetic acid
GFAAS: Graphite furnace atomic absorption spectrophotometry
GIS: Geographic information system
GN: “Grama Niladhari”-Public service officer
NTU: Nephelometric Turbidity Unit
SD: Standard Deviation
SLS: Sri Lanka Standards
TDS: Total Dissolved Solids
WHO: World Health Organization
