# Supplementary material for: Impact of water quality on Chronic Kidney Disease of unknown etiology (CKDu) in Thunukkai Division in Mullaitivu District, Sri Lanka
Source: BMC Nephrol. 2020 Nov 25;21:507. doi: 10.1186/s12882-020-02157-1 (PMC7687782; doi:10.1186/s12882-020-02157-1)
Supplement: Supplementary file 1 — Additional file 1. [file 12882_2020_2157_MOESM1_ESM.doc]

**Questionnaire**

Name of the person completing this form:………………………………….Date:……………

**QUESTIONNAIRE FOR CHRONIC KIDNEY DISEASE OF UNKNOWN ETIOLOGY *(*CKDu) IN MULLAITVU OF SRI LANKA**

Patient’s Name:……………………………………….. Date of Birth:……………………….

Sex……………………….Age……………………………..

Occupation…………………………………………

Address…………………………………………………………………………………………………………………………………………………………………………………………

1. How long have you been living in this address…………………………..
2. If you lived less than a year in the above address, give previous addresses..…………………………………………………………………………………………………………………………………………………………………………………………
3. Have you ever been told that you have a kidney disease? Y / N
4. How long has it been since you were first diagnosed?

(Circle one) < 1 year / 1-3 years / 3-5 years / 5-10 years / > 10 years

1. Average volume of drinking water per day:

1. ≤ 1 2. > 1 ≤ 2 L 3. > 2L

1. What is your source of drinking water? ……………………………………………
2. How long have you been using that source of water ………………………………
3. Are you drinking; Normal/ Hot/ Boiled cooled/ Filtered water?
4. Personal History

I currently smoke cigarettes, cigars, or pipes/chew tobacco. (Y/N)

I used to smoke cigarettes, cigars, or pipes/chew tobacco. (Y/N)

How many years did you smoke or chew tobacco?........................................................

I drink alcohol on a daily/weekly/monthly basis…………………………………….

I rarely drink alcohol(Y/N) ………………………………………………………..

I used to drink alcohol on a regular basis (Y/N) …………………………………..

I quit………………………years ago.

1. Do you have diabetics or hypertension?
2. If yes, how many years you are suffering from the disease?....................................
3. Patient’s serum creatinine amount.............................................Date obtained=………..
4. CHECKLIST FOR WATER SOURCE

| **Parapet wall** | | | |
| --- | --- | --- | --- |
| Does the well have a parapet wall? | Yes No | If yes, what is the height of the parapet wall? |  |

| **Impervious casing** | | | |
| --- | --- | --- | --- |
| Does the well have an impervious casing below the ground level? | Yes No | If yes, what is the height below the ground level? |  |

| **Platform / Apron** | | | |
| --- | --- | --- | --- |
| Does the well have a platform/ apron? | Yes No | If yes, what is the width of it? |  |
|  |  | Is it sloping outwards to allow water to drain away from well? |  |

| **Concrete cover on top** | | | |
| --- | --- | --- | --- |
| Does the well have a concrete cover on top? | Yes No | Does it have a manhole? |  |
|  |  | Is the rim of the manhole raised and for how much height? |  |

| **Drainage from well** | | | |
| --- | --- | --- | --- |
| Does the well have lead way drain from it? | Yes No | If yes, what is the length of it? |  |

18

| **Water withdrawal system** | | | |
| --- | --- | --- | --- |
| Does the well have a water withdrawal system? | Yes No | If yes, mention whether it is a water pump system or bucket? |  |

| **Other drainage system** | | | |
| --- | --- | --- | --- |
| Is there any other drainage system within 200 feet from the well? | Yes No | If yes, how far from well? |  |
|  |  | What type of drainage? |  |

| **Dumping places** | | | |
| --- | --- | --- | --- |
| Are there any dumping places within 50 feet from the well? | Yes No | If yes, how far from the well? |  |
|  |  | What type of dumping place? |  |
